# A model mimicking catabolic inflammatory disease; a controlled randomized study in humans

**DOI:** 10.1371/journal.pone.0241274

**Published:** 2020-11-05

**Authors:** Maike Mose, Nikolaj Rittig, Ulla Ramer Mikkelsen, Niels Jessen, Mads Bisgaard Bengtsen, Brit Christensen, Jens Otto Lunde Jørgensen, Niels Møller

**Affiliations:** 1 Department of Endocrinology and Internal Medicine, Aarhus University Hospital, Aarhus, Denmark; 2 STENO Diabetes Center, Aarhus University Hospital, Aarhus, Denmark; 3 Nutrition & Health, R&D, Arla Foods Ingredients Group P/S, Viby, Denmark; 4 Arla Foods amba, Aarhus, Denmark; University of Florida, UNITED STATES

## Abstract

**Objective:**

Inflammatory disease is catabolic and associated with insulin resistance, increased energy expenditure, lipolysis and muscle protein loss. The main contributors to these metabolic adaptations are inflammation, malnutrition and immobilisation. Controlled experimental models incorporating these central elements of hospitalisation are lacking. The aim of this study was to validate such a human experimental model.

**Methods:**

In a randomized crossover design, six healthy young men underwent; (i) overnight fast (CTR), or (ii) exposure to systemic lipopolysaccharide (1 ng/kg) combined with 36-hour fast and bed rest (CAT). The difference in insulin sensitivity between CAT and CTR was the main outcome, determined by a hyperinsulinemic euglycemic glucose clamp. Palmitate, glucose, urea, phenylalanine and tyrosine tracers were infused to estimate metabolic shifts during interventions. Indirect calorimetry was used to estimate energy expenditure and substrate oxidation.

**Results:**

Insulin sensitivity was 41% lower in CAT than in CTR (M-value, mg/kg/min): 4.3 ± 0.2 vs 7.3 ± 1.3, p<0.05. The median (min max) palmitate flux (μmol/min) was higher during CAT than in CTR (257.0 (161.7 365.4) vs 131.6 (92.3 189.4), p = 0.004), and protein kinetics did not differ between interventions. C-reactive peptide (mg/L) was elevated in CAT compared with CTR (30.57 ± 4.08 vs 1.03 ± 0.19, p<0.001). Energy expenditure increased by 6% during CAT compared with CTR (1869 ± 94 vs 1756 ± 58, p = 0.04), CAT having higher lipid oxidation rates (p = 0.01) and lower glucose oxidation rates (p = 0.03). Lipopolysaccharide caused varying abdominal discomfort 2 hours post-injection, which had disappeared the following day.

**Conclusion:**

We found that combined systemic inflammation, fasting and bed rest induced marked insulin resistance and increased energy expenditure and lipolysis, rendering this controlled experimental model suitable for anti-catabolic intervention studies, mimicking clinical conditions.

## Introduction

Hospitalisation is accompanied by muscle wasting and altered metabolism due to the catabolic combination of immobilisation, inflammation and calorie restriction [1–3]. Other catabolic features include insulin resistance and increased energy expenditure (EE), lipolysis and whole body protein turnover [2, 4–7].

Interventional studies investigating catabolic disease is limited by the substantial heterogeneity amongst patients. A number of studies have investigated patients in the intensive care units, but studies of more common and moderate disease conditions are generally lacking [8, 9]. Human controlled studies have used prolonged periods of fasting to explore the effects of low/non energy intake [5], bed rest to examine the catabolic effects of immobilization [6], and systemic lipopolysaccharide (LPS) exposure to investigate the acute catabolic effects of inflammation [4]. These models have focused on the isolated effects of these catabolic stimulants even though most catabolic states are caused by a combination of all three components. Furthermore, abdominal discomfort and nausea have precluded administration of oral interventions acutely after LPS-induced systemic inflammation.

At the cellular level, muscle catabolism involves a number of specific signalling events. Increased activation of muscle protein breakdown pathways (muscle RING-finger protein-1 (MURF1), F-Box protein 32 (Fbx32), Unc-51 like autophagy activating kinase 1 (ULK1), and nucleoporin P62 (P62)) may be involved in muscle wasting together with decreased activation of the muscle protein synthesis pathways (Protein kinase B (Akt) and mammalian target of rapamycin (mTOR)) [10–13]. Again, very little is known about the muscle signalling responses in the initial phase of catabolic conditions.

The present study was designed to validate and define the metabolic impact and tolerability of a human model, combining the late phase of LPS-induced systemic inflammation, immobilisation (bed rest) and fasting. Such a human model could be valuable in future anti-catabolic intervention studies.

## Material and methods

### Participants

**Fig 1:**
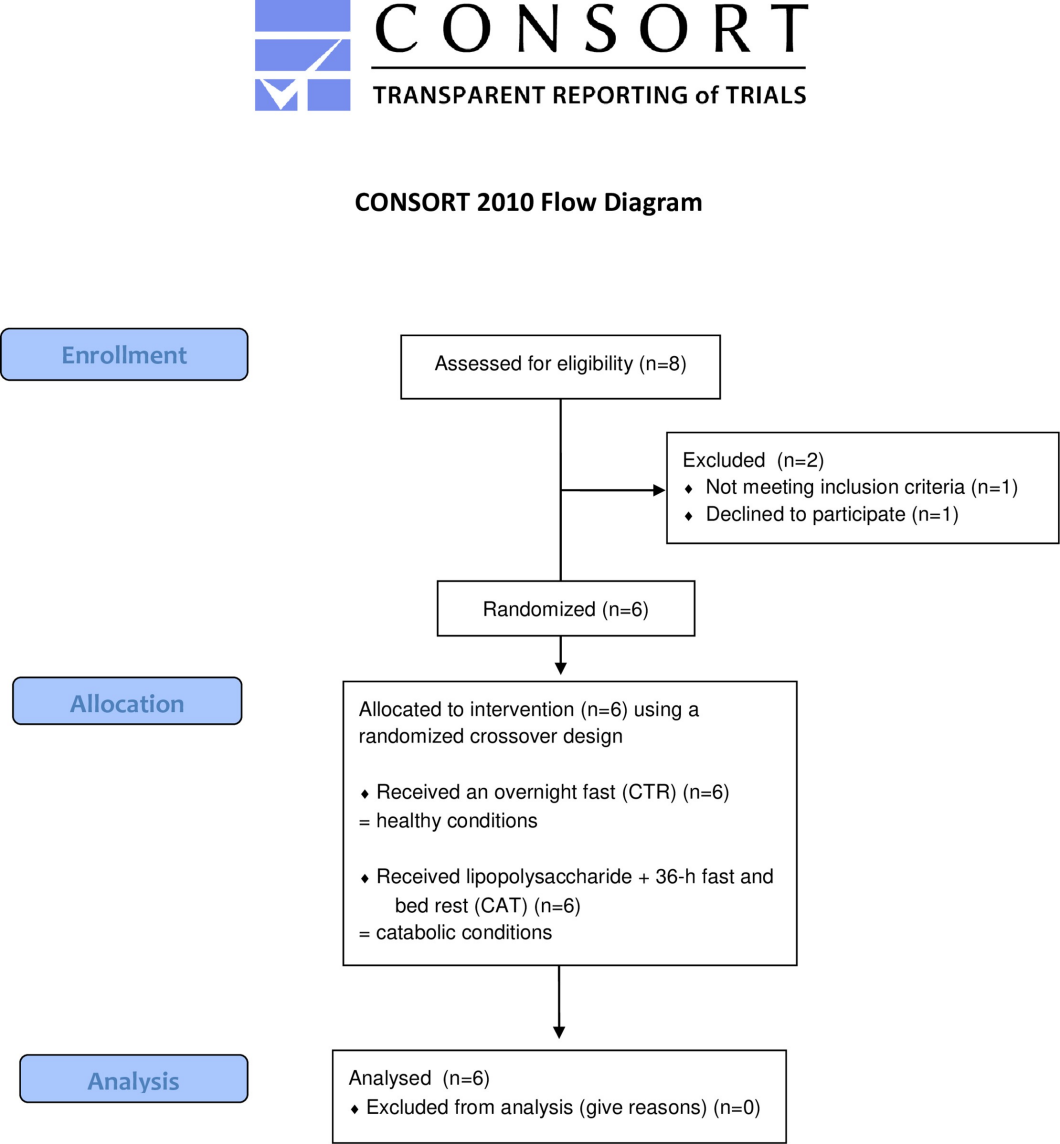
Consort flow diagram. Consort flow diagram of the study.

Participants were eligible for inclusion if they were healthy males without regular intake of medication, between 20 to 40 years of age, had a body mass index (BMI) between 20–30 kg*m^-2^ and were non-smokers. Screening of participants involved a physical examination and medical interview including an electrocardiogram and a blood-screen-test. Participants were excluded if abnormal test results were recorded. A consort flowchart is shown in Fig 1. All studies were performed at our research unit, Department of Endocrinology and Internal Medicine, Aarhus University Hospital (Denmark). Participants were without febrile illness the preceding week before each study. They did not exercise 48 hours prior to the visit and were instructed to eat a normal meal the night before the study (protein 10–20%, fat max 30%, carbohydrates 50–60%). All participants arrived by taxi at 7.00 AM after an overnight fast.

### Ethics

The study was approved by The Danish Ethical Committee (1-10-71-41-17), registered at clinical trials (NCT03158363), and both written and oral consent was obtained prior to inclusion. The study was conducted in line with good clinical practice guidelines and followed the principles of the Declaration of Helsinki. Shortly, participants were thoroughly informed about any discomfort and possible side effects associated with the experimental procedures, and that consent to participate could be withdrawn at any time point prior to or during the experiments.

### Interventions

We used a randomized crossover design to investigate six healthy, lean male participants on two occasions separated by at least four weeks:

- LPS-induced inflammation in combination with a 36-hour fast and bed rest (CAT)
- Overnight fast as a control (CTR).

**Fig 2:**
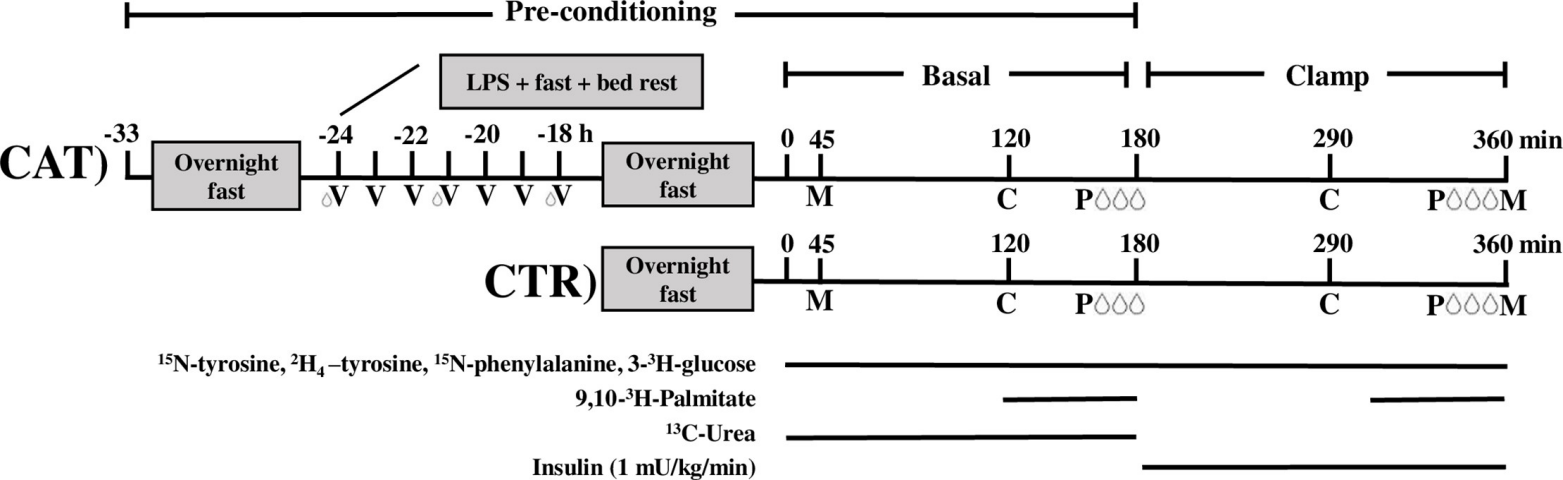
Design of the study. CAT (pre-conditioning with lipopolysaccharide) (LPS, 1 ng/kg) exposure (inflammation) + 36 hour bed rest and fast, CTR (pre-conditioning with an overnight fast), vital parameters (V), calorimetry (C), plethysmography (P), blood sample (droplet) and muscle biopsy (M).

The CAT study day was preceded by pre-conditioning with a total of 36 hours of fasting (only tap-water allowed) and bed rest combined with LPS infusion (t = -24 h). Blood pressure, temperature and heart rate were measured hourly for 6 hours following LPS-exposure (t = -24 to -18 h) (Fig 2), where after participants were kept overnight at the Medical ward to ensure adherence to the bed rest and fasting protocol. Participants were randomized using a computerised randomization system by the primary investigator.

During the study day (t = 0–360 min), a catheter was inserted into the cubital vein for infusion of tracers and into a dorsal hand vein for blood sampling. The dorsal hand vein was heated to collect arterialized blood [14]. Further, a retrograde catheter in the deep cubital contralateral vein was inserted for deep venous blood sampling. The study day consisted of a 3-hour basal period (included in the total of 36 h of fasting and bed rest) and a 3-hour hyperinsulinemic euglycemic clamp (HEC) period. Indirect calorimetry measurements and muscle biopsies were performed during both periods (Fig 2).

### Outcomes

The primary outcome was insulin sensitivity between CTR and CAT quantified by HEC. Secondary outcomes were metabolic shifts in glucose, lipid and protein metabolism, and changes in hormones, substrates and intramyocellular signalling between CTR and CAT. Sample size was determined by a pre-study power calculation on the primary outcome.

### Lipopolysaccharide

A bolus (1 ng/kg) of E. coli lipopolysaccharide (10,000 USP Endotoxin, lot HOK354; The United States Pharmacopeia Convention, Inc., Rockville, Maryland) was infused intravenously over two minutes 24 hours prior to the study day (t = -24 h) and followed by 10 ml saline infusion.

### Hyperinsulinemic euglycemic clamp

Following the basal period of both interventions a 3-hour insulin-stimulated HEC period (t = 180–360 min, Fig 2) was initiated using a constant insulin infusion rate of 1 mU/kg/min (Insulin Actrapid; Novo Nordisk, Copenhagen, Denmark). Plasma glucose concentrations were maintained at 5 mmol/L by variable infusion rates of 20% glucose, and glucose concentrations were measured on-site every 10 minutes throughout the HEC. Insulin sensitivity, expressed as M-value (mg/kg/min), was calculated when steady state was reached at the end of the HEC. M-value = Infusionrate(glucose 200 g/L)*[glucose (mmol/L)] / total body weight (kg).

### Indirect calorimetry

Respiratory gases were collected for 15 minutes using an Oxycon Pro calorimeter (Intramedic, Denmark) with a canopy at t = 120 and t = 290 min (Fig 2). The energy expenditure (EE), respiratory exchange rate (RER), and oxidation rates of glucose and lipids were calculated as described [15]. Urine was collected in order to measure nitrogen excretion rates and calculate protein oxidation rates.

### Protein, glucose and palmitate kinetics

At t = 0 a primed continuous infusion of ^13^C-Urea (prime 390.6 mg, infusion rate 42 mg/h), L-ring-^2^H_4_-tyrosine (prime 0.6 mg/kg, infusion rate 0.6 mg/kg/h), ^15^N-phenylalanine (prime 0.75 mg/kg, infusion rate 0.75 mg/kg/h), 3-^3^H-glucose (prime 20μCI, infusion rate 12 μCi/min) and bolus ^15^N-tyrosine (prime 0.3 mg/kg) were started and continued throughout the study. 9,10-^3^H-palmitate (infusion rate 0.3 μCi/min) was infused between t = 120–180 and t = 290–350 min (Fig 2).

Protein-, palmitate- and glucose kinetics were calculated as previously described [16, 17].

Isotopic tracer enrichment in the blood samples were drawn in triplicates during the basal period (t = 160–180 min) and analysed using gas chromatography mass spectrometry. Arteriovenous blood samples and blood flow (venous occlusion plethysmography [18]) across the forearm muscle were collected at the end of the basal- and the HEC period (Fig 2).

### Blood sample analysis

Blood samples were stored at -20°C and analysed in batches following study completion. Plasma glucagon concentrations were analysed using a radioimmunoassay (EMD Millipore's Glucagon Radioimmunoassay (RIA) Kit, Germany), plasma glucose concentrations were measured using YSI 2300 model Stat Plus (Bie & Berntsen, Denmark), serum insulin concentrations were analysed using ELISA (Mercodia, Sweden), and free fatty acids (FFA) were analysed using in vitro enzymatic colorimetric method assay NEFA-HR(2) (Wako Chemicals GmbH, Germany).

### Muscle biopsies

With sterile procedure in local anaesthesia (10 mL lidocaine), a Bergström´s needle was used to collect biopsies from vastus lateralis of the quadriceps muscle at t = 45 min and t = 360 min. Biopsies were immediately snap-frozen in liquid nitrogen and stored at -80⁰C until analysed.

Before Western blotting, biopsy samples were homogenized in a buffer with a 7.4 pH-level and contained 50 mM HEPES, 10 mM Na_4_P_2_O_7_, 20 mM NaF, 137 mM NaCl, 5 mM EDTA, 5 mM NAM, 10 mM TSA, 1 mM MgCl_2_, 1 mM CaCl_2_, 2 mM NaOV, HALT protease inhibitor cocktail 1%, 1% NP-40, and 10% glycerol. Samples were centrifuged at 14,000 g for 20 min. Control for equal loading was assessed by the stain free technology [19]. Image Lab 5.0, Bio-Rad laboratories, was used for visualisation and quantification. The primary antibodies used were Akt (total #4691, ser473 #9271; Cell signalling), mTOR (total #2972, ser2448 #5536; Cell signalling), S6 (p70S6 kinase, total #2217, ser235/236 #4858; Cell signaling), 4EBP1 (Eukaryotic translation initiation factor 4E-binding protein 1, total #9644), non-phosphorylated 4EBP1 (thr46 #4923; Cell signaling), Fbx32 (total #ab92281; Abcam), ULK1 (total #4773, ser757 #6888, ser555 #5869; Cell signalling), MURF1 (total #ab172479; Abcam), and p62 (total #ab56416; Abcam). Results are expressed as the ratio between phosphorylated protein and total protein measurements, unless stated otherwise. For each phosphorylated target, it was checked that interventions did not change total protein levels.

### Statistics

For graphs and statistical analysis, Sigma Plot 11 (San Jose, California, USA) and STATA 13 (College Station, Texas, USA) were used. QQ-plots of residuals were inspected. If data showed variance heterogeneity, logarithmic transformation was used. Repeated-measures mixed model was used with intervention and time as factors (and any interaction between them). Multiple post hoc Bonferoni corrected t-tests were performed, when interaction was present (p<0.05). For parameters only measured twice, paired t-test was used, or non-parametric test when the criteria of normal distribution was not met. Data are presented as means ± SEM, medians (min max) or mean difference [95% CI]. P-values < 0.05 were considered significant.

## Results

### Subjects

The study was conducted between June and September 2017. All participants completed the study and were included in the analysis (Fig 1). Baseline characteristics are shown in Table 1. There were no adverse events related to the interventions. Participants experienced varying degrees of nausea, headache, shivering and muscle-pain in the hours following LPS-injection during pre-conditioning in the CAT group. All symptoms had disappeared on the study day.

**Table 1:** Baseline charateristics.

Demographic data		
Age	*years*	24 (20 29)
BMI	*kg/m*^*2*^	25 (23 28)
Body weight	*kg*	79 (66 89)
Height	*cm*	177 (169 184)
**Clinical data**	* *	
P-Cholesterol	*mmol/L*	4.4 (3.6 5.8)
P-Alanine transaminase	*U/L*	41.5 (15 65)
Hemoglobin A1c	*mml/mol*	32.5 (28 37)
P-Thyroid stimulating hormone	*10*^*3*^*/L*	2.18 (0.43 3.22)
P-Creatinine	*umol/L*	78 (65 98)
P-CRP	*mg/L*	1.9 (0.6 3.5)
B-Leucoytes	*10*^*9*^*/L*	6.43 (4.98 7.18)
B-Haemoglobin	*mmol/L*	9.45 (8.3 10.2)
B-Trombocytes	*10*^*9*^*/L*	249 (186 298)

Baseline characteristics (median (range)) of the six participants in the study.

### Inflammatory biomarkers

Plasma concentrations of C-reactive peptide (CRP) were significantly increased during CAT compared with CTR, whereas plasma concentrations of leucocytes were not significantly different (Table 2). Data during pre-conditioning of CAT acutely after LPS exposure (t = -24 h to -18 h) caused a significant elevation in core temperature, heart rate, circulating leucocytes, tumor necrosis factor alpha (TNFα) and interleukin-6 (IL-6) (S1 Fig).

**Table 2:** Hormones, substrates and indirect calorimetry.

Hormones and substrates		* *	CTR	CAT	CTR >< CAT	basal >< clamp
Leucocytes	basal	*x 10*^*9*^*/L*	5.14 ± 0.20	5.89 ± 0.49	p = 0.19	** **
C-reactive peptide	basal	*mg/L*	1.03 ± 0.19	30.57 ± 4.08	p<0.0001	** **
Glucose	basal	*mmol/L*	5.0 (4.70 5.27)*	4.37 (3.95 4.67)^#^	p < 0.0001	*p = 1.0, ^#^p < 0.0001
	clamp	* *	5.0 (4.6 5.27)	4.98 (4.47 5.07)	p = 1.0	interaction p = 0.009
A-V net balance_glu_ x flow	basal	*μmol/100 mL/min*	0.28 ± 0.14*	0.07 ± 0.10^#^	p = 1.0	*p < 0.0001, ^#^p = 0.39
	clamp	* *	2.73 ± 0.52	0.54 ± 0.23	p < 0.0001	interaction p = 0.0019
Insulin	basal	*pmol/L*	27.3 (18 49)	17.5 (12.0 31.5)	p = 0.045	p < 0.0001
	clamp	* *	342.8 (291 451)	312.8 (256.0 367.5)		
Glucagon	basal	*pmol/L*	7.47 (4.94 18.7)*	17.2 (8.4 25.5)^#^	p = 0.07	*p < 0.0001, ^#^p < 0.0001
	clamp	* *	3.68 (2.35 9.50)	3.23 (2.03 6.41)	p = 1.0	
Growth hormone	basal	*ng/mL*	0.13 (0.06 1.10)*	1.12 (0.19 9.23)^#^	p = 0.03	*p = 0.33, ^#^p = 0.02
	clamp	* *	0.30 (0.07 2.93)	0.20 (0.10 0.46)	p = 0.38	interaction p = 0.03
Lactate	basal	*mmol/L*	0.89 ± 0.17	0.73 ± 0.06	p = 0.16	p = 0.06
	clamp	* *	1.05 ± 0.08	0.93 ± 0.18		
Adrenaline	basal	*pg/mL*	44.5 ± 11.9	52.7 ± 9.8	p = 0.25	p = 0.24
	clamp	* *	51.9 ± 11.5	57.0 ± 10.9		
Noradrenaline	basal	*pg/mL*	185.2 ± 16.5	208.3 ± 46.2	p = 0.14	p = 0.35
	clamp	* *	183.3 ± 22.5	237.7 ± 28.3		
Cortisol	basal	*ng/mL*	100.0 ± 23.8	108.9 ± 18.6	p = 0.65	p = 0.79
Free fatty acids, FFA	basal	*mmol/L*	0.32 (0.22 0.36)	0.70 (0.42 0.99)	p = 0.002	p <0.0001
	clamp	* *	0.02 (0.01 0.04)	0.05 (0.03 0.12)		
βhydroxybutyrate	basal	*umol/L*	100 ± 0 (detection limit)	955.3 ± 198.8	p = 0.002	
**Calorimetry**		* *				
Energy Expendiure, EE	basal	*kcal/day*	1753 ± 58*	1869 ± 94^#^	p = 0.04	* p = 0.15, ^#^p = 0.01
	clamp	* *	1832 ± 79	1737 ± 72	p = 0.10	interaction p = 0.004
Glucose oxidation	basal	*mg/kg/min*	1.67 ± 0.26	0.60 ± 0.16	p = 0.03	p = 0.004
	clamp	* *	2.72 ± 0.51	1.32 ± 0.25		
Lipid oxidation	basal	*mg/kg/min*	0.56 ± 0.07	1.14 ± 0.05	p = 0.01	p = 0.001
	clamp	* *	0.21 ± 0.17	0.74 ± 0.09		
Protein oxidation	basal	*mg/kg/min*	0.79 ± 0.03	0.75 ± 0.06	p = 0.57	

* = different from clamp within CTR (p<0.05)

# = different from clamp within CAT (p<0.05)

Data are depicted as mean ± SEM or median (range) for CTR (overnight fast) and CAT (Inflammation + 36 hour fast and bed rest) measured at the end of the basal (insulin -) and hyperinsulinemic euglycemic clamp period (insulin +). Comparisons were made using repeated-measures mixed model with intervention and time as factors (and any interaction between them), and post hoc Bonferroni corrected t-tests were used to compare groups. A paired t-test was used to compare urea flux (only measured in the basal period).

### Insulin sensitivity

**Fig 3:**
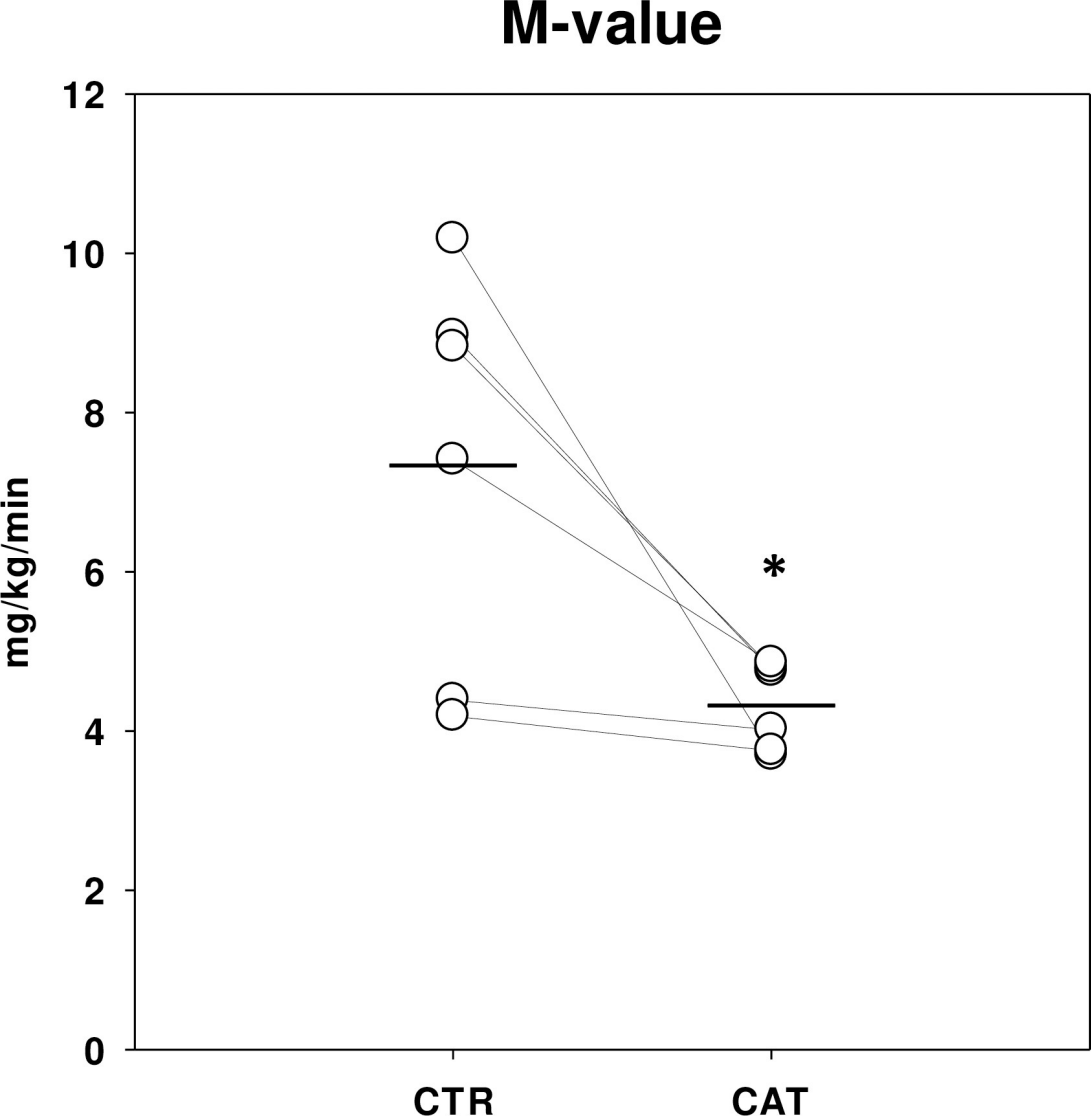
Hyperinsulinemic euglycemic clamp. M-value (mg/kg/min) for CTR (overnight fast) and CAT (Inflammation + 36 hour bed rest and fast) are depicted with paired individual data points (white dots connected by a straight line) and means (horizontal lines). Analysed with paired t-test, p-values < 0.05 were considered significant (*). N = 6.

During insulin stimulation (HEC), insulin sensitivity (M-value) was reduced by 41% after CAT compared with CTR (Fig 3), reflecting peripheral insulin resistance. Endogenous glucose production (EGP) was completely suppressed in both CTR and CAT during insulin stimulation (Table 3). CAT induced slightly lower insulin levels than in CTR at baseline and during the insulin-stimulated period (Table 2), whereas glucagon, growth hormone and β-hydroxybutyrate were elevated in CAT compared with CTR at baseline (Table 2). The arteriovenous difference of glucose across the forearm muscle (A-V net balance_glu_ x flow) increased more in CTR compared with CAT during insulin stimulation, whereas baseline values in CTR and CAT were similar (Table 2). Blood flow was significantly higher in CTR than in CAT (Table 3).

**Table 3:** Protein, glucose and lipid kinetics.

Whole body protein metabolism		* *	CTR	CAT	CTR >< CAT	basal >< clamp
Urea flux	basal	*μmol/kg/h*	286.8 (219.7 318.86)	264.8 (209.0 445.0)	p = 0.66	
Phenylalanine conversion	basal	*umol/kg/h*	3.54 ± 0.89	2.70 ± 0.71	p = 0.47	p = 0.51
Qphe -> Qtyr	clamp	* *	3.18 ± 0.25	2.34 ± 0.92		
Protein breakdown	basal	*umol/kg/h*	37.9 ± 1.57	40.0 ± 1.05	p = 0.55	p <0.0001
endoQphe	clamp	* *	33.0 ± 1.97	33.3 ± 1.04		
Protein synthesis	basal	*umol/kg/h*	34.3 ± 2.52	37.3 ± 1.24	p = 0.45	p <0.0001
Qphe—(Qphe -> Qtyr)	clamp	* *	29.3 ± 2.43	31.0 ± 1.38		
Protein netbalance	basal	*umol/kg/h*	-3.54 ± 0.89	-2.70 ± 0.71	p = 0.47	p = 0.51
synthesis—breakdown	clamp	* *	-3.18 ± 0.25	-2.34 ± 0.92		
**Forarm muscle metabolism**	** **	* *				
Muscle breakdown, Ra_phe_	basal	*μg/100 mL/min*	6.46 ± 1.37	9.99 ± 2.62	p = 0.42	p = 0.01
	clamp	* *	3.99 ± 0.66	3.28 ± 0.91		
Muscle synthesis, Rd_phe_	basal	*μg/100 mL/min*	4.50 ± 0.10	7.56 ± 2.63	p = 0.47	p = 0.08
	clamp	* *	3.41 ± 0.60	2.73 ± 0.84		
Muscle netbalance, NBphe	basal	*μg/100 mL/min*	1.96 ± 0.53	2.43 ± 0.68	p = 0.67	p = 0.006
	clamp	* *	0.58 ± 0.40	0.55 ± 0.27		
Forearm blood flow	basal	*mL/100 mL/min*	3.19 (2.95 6.09)	2.23 (1.64 2.76)	p = 0.04	p = 0.33
	clamp	* *	2.75 (2.01 3.94)	2.45 (1.61 3.49)		
**Fat metabolism**	** **	* *				
Palmitate flux	basal	*umol/min*	131.6 (92.3 189.4)	257.0 (161.7 365.4)	p = 0.004	p < 0.0001
	clamp	* *	29.0 (22.2 61.6)	54.3 (31.3 70.2)		
**Glucose metabolism**	** **	* *				
Endogen glucose production, EGP	basal	*mg/kg/min*	1.56 ± 0.07	1.34 ± 0.10	p = 0.11	p < 0.001
	clamp	* *	0.22 ± 0.28	0.31 ± 0.20		
Glucose Rd	basal	*mg/kg/min*	1.62 (1.54 2.09)*	1.47 (1.18 1.64)^#^	p = 0.88	*p < 0.0001, ^#^ p < 0.0001
	clamp	* *	8.45 (4.40 10.6)	3.90 (3.18 4.47)	p< 0.0001	interaction p = 0.007

* = different from clamp within CTR (p<0.05)

# = different from clamp within CAT (p<0.05)

Metabolic tracer data from CTR (overnight fast) and CAT (Inflammation + 36 hour fast and bed rest) measured at the end of the basal (insulin -) and hyperinsulinemic euglycemic clamp period (insulin +). Data are depicted as mean ± SEM or median (min max), and were analysed using repeated-measures mixed model with intervention and time as factors (and any interaction between them) and post hoc Bonferroni t-tests were used to compare groups. Parameters only measured in the basal period was analysed using paired t-test. (-) = not measured.

### Fat metabolism

CAT increased lipolysis (palmitate flux) and free fatty acid (FFA) concentrations compared with CTR (Tables 2 and 3). Insulin stimulation reduced lipolysis (palmitate flux) during both conditions, but was not reduced to the same absolute level during CAT and CTR (Table 3).

### Protein metabolism

Whole body protein metabolism was unaltered in CAT compared with CTR, and urea fluxes were similar in the two settings. No difference was observed between CAT and CTR regarding muscle protein rate of appearance (Ra_phe_, breakdown), rate of disappearance (Rd_phe_, synthesis) or net balance (NB_phe_) in the basal period (Table 3). Insulin stimulation decreased muscle protein breakdown with no effect on protein synthesis, leading to significantly increased forearm NB_phe_ in both CTR and CAT with no difference between groups (Table 3).

### Indirect calorimetry

EE was 6% higher during CAT than in CTR during the basal period (Table 2). Glucose oxidation rates were lower and lipid oxidation rates were higher during CAT conditions than during CTR conditions in the basal period. The HEC increased glucose oxidation rates and decreased lipid oxidation rates in both CAT and CTR (Table 2). We found no difference in protein oxidation rates between CTR and CAT in the basal period (Table 2).

### Skeletal muscle signaling

**Fig 4:**
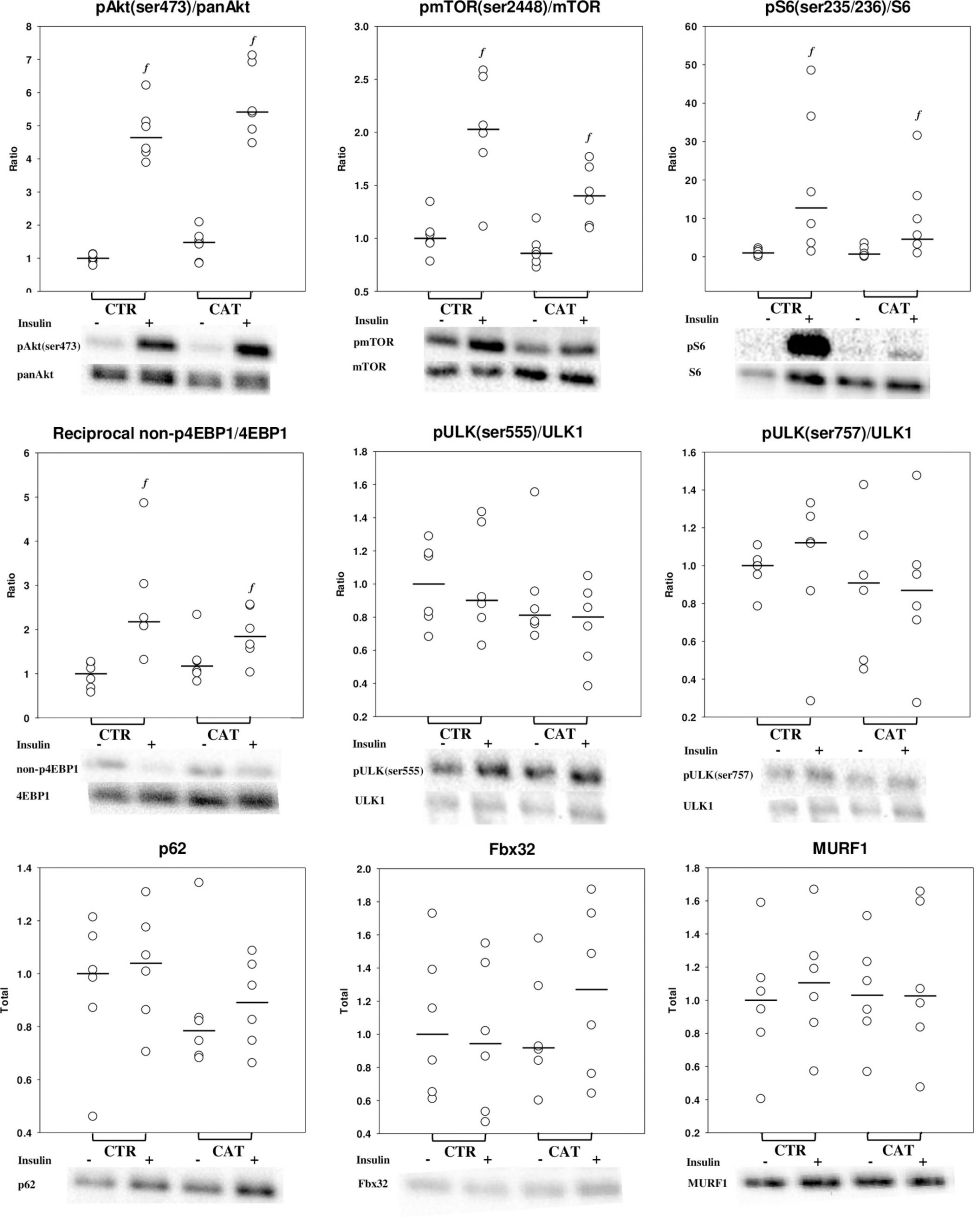
Intramyocellular signaling. Representative Western blots of muscle biopsies during the basal (insulin -) and hyperinsulinemic euglycemic clamp period (insulin +). Interventions CTR (overnight fast) and CAT (Inflammation + 36 hour bed rest and fast) are depicted with individual data points (white dots) and medians (horizontal lines). Analysed using repeated-measures mixed model with Bonferroni post hoc corrections. *f* = significantly different from the basal period within the same intervention (p<0.05). mTOR = mammalian target of rapamycin, S6 = p70S6 kinase, 4EBP1 = Eukaryotic translation initiation factor 4E-binding protein 1 and ULK1 = Unc-51 like autophagy activating kinase 1, P62 = Nucleoporin P62, FBX32 = F-Box protein 32 and MURF1 = Muscle RING-finger protein-1. N = 6.

We found no difference in activation of muscle signalling in the synthesis or breakdown pathways between CAT and CTR (Fig 4). Insulin stimulation increased pAkt(ser273)/Akt (median ratio [95%CI]; 4.5-fold [3.8 5.3], p<0.0001, interaction p = 0.41), pmTOR(ser2448)/mTOR (median ratio [95%CI]; 1.7-fold [1.5 2.0], p<0.0001, interaction p = 0.09) and reciprocal non-p4EBP1/4EBP1 (median ratio [95%CI]; 2-fold [1.4 2.7], p<0.001, interaction p = 0.05) equally in CTR and CAT (Fig 4). Total mTOR significantly decreased during insulin stimulation (p = 0.02), which could overestimate the effects of HEC on pmTOR/mTOR. Insulin stimulation did not alter any muscle breakdown targets (pULK(ser555)/ULK1, pULK(ser757)/ULK1, p62, MURF1 or Fbx32, all p`s>0.05).

## Discussion

This study was designed with the purpose of establishing a novel experimental catabolic model by combining LPS administration with 36 hours of bed rest and fasting. Our main findings are that the CAT-model generates marked insulin resistance, increases EE, lipolysis and lipid oxidation without affecting protein fluxes. This controlled human model was safe and imitates catabolic conditions occurring in a clinical setting (i.e. febrile illness).

We found a significant 41% decrease in insulin sensitivity in CAT compared with CTR. Prolonged bed rest, fasting and acute inflammation are all known to induce insulin resistance. A previous study has shown how a combination of a 24-hour bedrest and “quasi”-fasting (No oral food-intake, but 200 kcal intravenous glucose infusion) reduced insulin sensitivity by 22% assessed by HEC in an otherwise healthy study population similar to ours [20]. A model applying prolonged bed rest (1 week) alone has shown a 29% reduction in insulin sensitivity compared with control conditions [6]. We have previously shown that insulin sensitivity is lowered by 35% in healthy young males in the hours following LPS exposure [4] and prolonged fasting (72 hours) induced a 59% decrease in insulin sensitivity [21]. In our CAT-model we combined LPS exposure, fasting, and bed rest to imitate a typical clinical infectious episode. Even though our investigations were performed 24 hours following LPS exposure and used shorter periods of fasting and immobilization compared with previous studies in the field, this model seem to cause a marked, sustained degree of insulin resistance.

Insulin levels were 9% lower during the insulin-stimulated period after CAT than in CTR even though the same dose of insulin/kg total bodyweight was given during both interventions. The insulin lowering effect of LPS has been found by others during HEC conditions in healthy male subjects [4], and may reflect increased metabolic clearance rate of insulin. This may contribute modestly to the observed insulin resistance, but it is not conceivable that the minute reduction in insulin levels acts significantly to the decrease in insulin sensitivity. Our data suggest that both muscle and fat become insulin resistant during CAT compared with CTR.

Insulin resistance in general is not restricted to glucose metabolism, but also induces increased lipolysis in adipose tissue and increased muscle protein loss *per se* [11]. In the present study our forearm data showed comparable negative rates of NB_phe_ (muscle net balance), Rd_phe_ (muscle synthesis) and Ra_phe_ (muscle breakdown) during CTR vs CAT, although inflammation, fasting and bed rest are believed to have a negative impact on muscle net balances in general [2, 3, 11, 22, 23]. However, most studies focusing on protein turnover during acute illness have investigated intensive care patients, which are heterogeneous and in general not representative of patients with more “common illness” in whom data on protein turnover are sparse [3, 8, 23].

In our hands insulin stimulation was anabolic in both CTR and CAT primarily by inhibiting breakdown (Ra_phe_), as reported by others [4, 11, 16]. In discrepancy, insulin stimulation promoted activation in muscle protein synthesis targets rather than inhibition of breakdown targets. This could relate to anatomic metabolic differences at the sampling sites (forearm versus thigh muscle), or reflect that activation of intramyocellular synthesis signalling pathways does not necessarily translate into increased muscle protein synthesis. Supporting the later, others have found insulin to stimulate muscle protein synthesis target phosphorylation without changing muscle protein synthesis [4, 24], possibly by a mechanism involving loss of mass action and hypoaminoacidemia caused by insulin-mediated inhibition of muscle protein breakdown [24]. Finally, protein metabolism was studied in the post-absorptive state without any amino acid supplementation; there are suggestions that a blunted response to anabolic stimuli (e.g. amino acids), known as anabolic resistance, might be an important single factor in disease-induced muscle loss [10, 25]. Future experiments supplementing participants with e.g. amino acids in our catabolic model, could contribute to the knowledge gap of whether ‘anabolic resistance’ plays an important role in muscle wasting during the initial phase of disease.

EE increases during many catabolic diseases [7, 23]. We found that CAT caused a 6% increase in EE compared with CTR. Others have shown how an isolated 24-hour fast caused a reduction in EE of 8.5% [26], whereas the combination of fast and bedrest left EE unchanged [20]. LPS is known to increase EE acutely in the hours following injection [4], whereas more prolonged effects on EE lack investigation. In this study, EE might be elevated due to the ongoing production of liver-mediated acute phase reactants and/or increased thermogenesis in the late phase of LPS-induced fever and inflammation. Surprisingly, we found whole body protein fluxes to be unaltered between CTR and CAT. Bearing in mind that the study was not powered for this outcome, this finding suggests that the overall impact of LPS on protein metabolism is waning and that redistribution of amino acids (for e.g. liver-mediated acute phase reactants) plays only a minor role 24 hours post LPS exposure, whereas whole body amino acid fluxes are known to increase acutely in the hours following LPS exposure [4]. Similar to our findings, a 72 h fast in lean healthy participants showed unaltered whole body protein fluxes [27]. Thus, the model incorporates catabolic features in terms of both increased energy demands (increased EE) and decreased energy availability (fasting). The hormonal stress response with increased glucagon and growth hormone and decreased insulin concentrations, contribute to increased lipolysis, ketogenesis and insulin resistance.

We studied the late phase of inflammation and, unlike other LPS-studies [4, 16], the cytokine concentrations and accompanying gastrointestinal symptoms experienced by our participants had therefore peaked earlier during pre-conditioning (S1 Fig) and gastrointestinal symptoms were not present on the study day. Therefore, this model appears both tolerable and suitable for controlled oral intervention studies in a catabolic inflammatory setting, without major limitations due to abdominal discomfort.

Our study has limitations. First, our study was conducted in younger males and our results may not necessarily apply to the frail elderly or to women. Secondly, the sample size was defined by a pre-study power calculation for the primary endpoint (insulin sensitivity), and some of our secondary findings may be prone to type 2 errors.

In conclusion, combining systemic inflammation, bed rest and fasting is catabolic and induce insulin resistance, increases EE, and accelerates lipolysis and lipid oxidation. This catabolic model may serve as a useful and clinically relevant tool in future intervention studies, and the absence of gastrointestinal side effects allows oral intervention studies.

## Supporting information

S1 Fig Vital parameters and inflammatory markers during pre-conditioning of CAT. Vital parameters (heart rate, axillary temperature and mean arterial blood pressure) and inflammatory markes following LPS exposure (t = -24 h) during pre-conditioning in CAT (LPS-induced inflammation + 36 h fast and bedrest), n = 6. Mean arterial pressure = 2/3 * systolic pressure + 1/3 * diastolic pressure. TNFα = tumor necrosis factor alpha. IL-6 = interleukine 6.
(TIF)

S1 Raw images (PDF)
