# Supplementary figures and images for: A model mimicking catabolic inflammatory disease; a controlled randomized study in humans
Source: PLoS One. 2020 Nov 5;15(11):e0241274. doi: 10.1371/journal.pone.0241274 (PMC7644057; doi:10.1371/journal.pone.0241274)

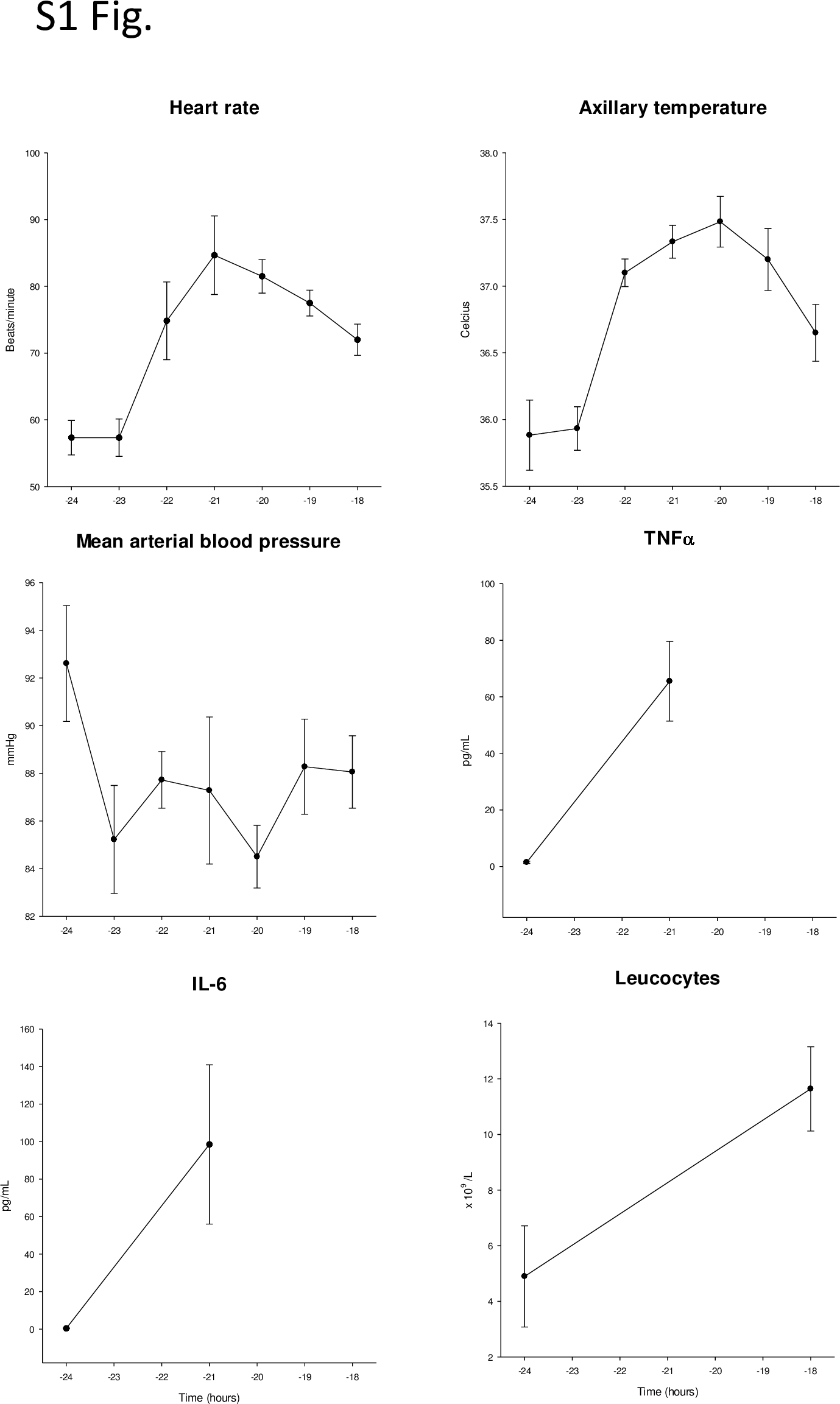

Supplement: S1 Fig — Vital parameters (heart rate, axillary temperature and mean arterial blood pressure) and inflammatory markes following LPS exposure (t = -24 h) during pre-conditioning in CAT (LPS-induced inflammation + 36 h fast and bedrest), n = 6. Mean arterial pressure = 2/3 * systolic pressure + 1/3 * diastolic pressure. TNFα = tumor necrosis factor alpha. IL-6 = interleukine 6. (TIF) [file pone.0241274.s001.tif]
