# Supplementary material for: A model mimicking catabolic inflammatory disease; a controlled randomized study in humans
Source: PLoS One. 2020 Nov 5;15(11):e0241274. doi: 10.1371/journal.pone.0241274 (PMC7644057; doi:10.1371/journal.pone.0241274)

S1_raw_images

In Fig 4 of the manuscript, mirrored images of id5 are displayed (in the order left to right: 21, 20, 19, 18) except for pS6 and S6 where id1 is displayed (in the order left to right: 1, 2, 3, 4)

The sample id is shown with red on the next page and is identical for all blots.

The interventions for each sample id is shown on the right.

Order of samples:

1: id1 CTR basal
2: id1 CTR clamp
3: id1 LPS basal
4: id1 LPS clamp
5: id2 LPS basal
6: id2 LPS clamp
7: id2 CTR basal
8: id2 CTR clamp
9: *internal control*
10: id3 CTR basal
11: id3 CTR clamp
12: id3 LPS basal
13: id3 LPS clamp
14: id4 CTR basal
15: id4 CTR clamp
16: id4 LPS basal
17: id4 LPS clamp
18: id5 LPS basal
19: id5 LPS clamp
20: id5 CTR basal
21: id5 CTR clamp
22: id6 LPS basal
23: id6 LPS clamp
24: id6 CTR basal
25: id6 CTR clamp
26: *internal control*

pAKT_raw_image

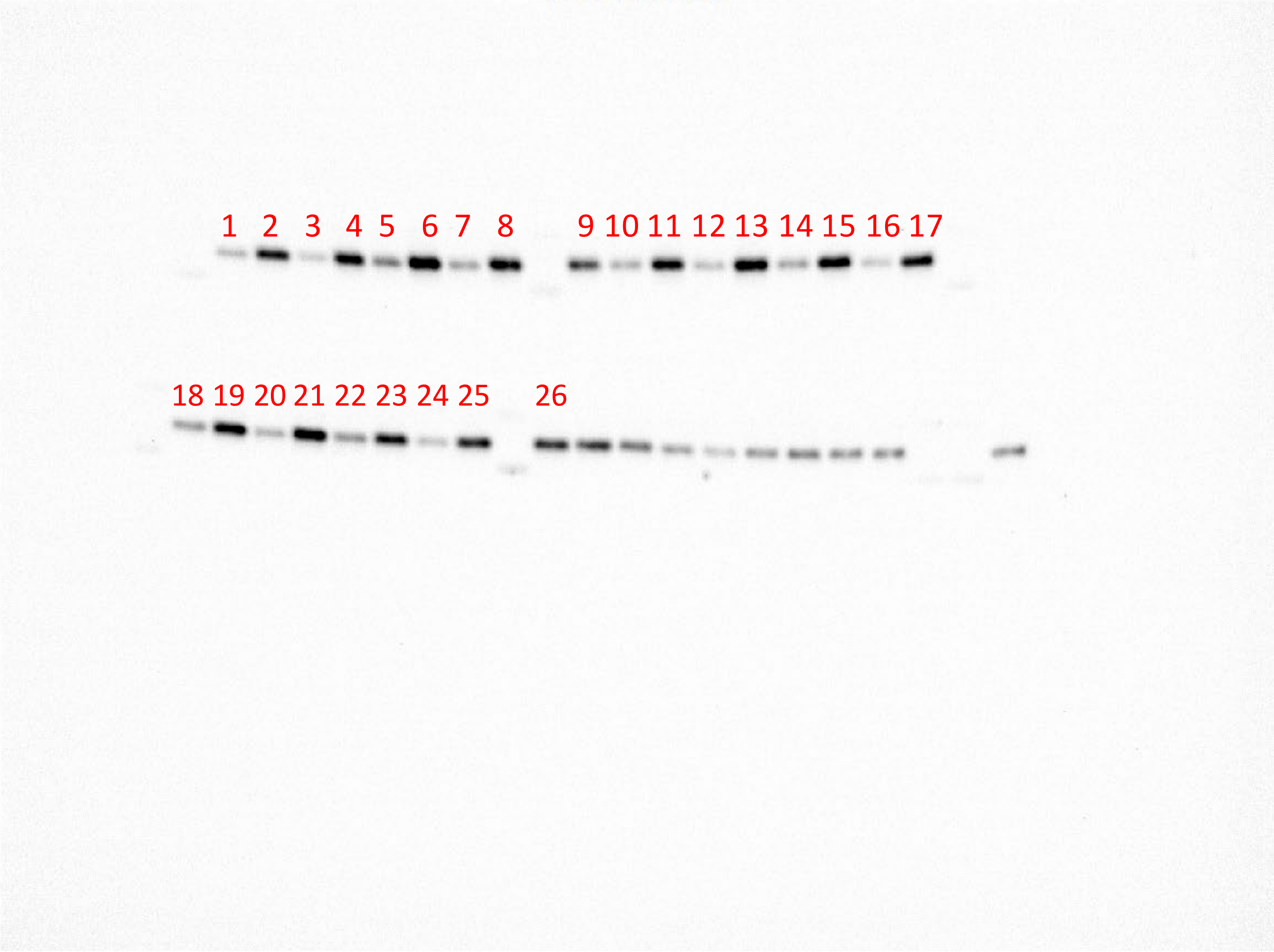

AKT_raw_image

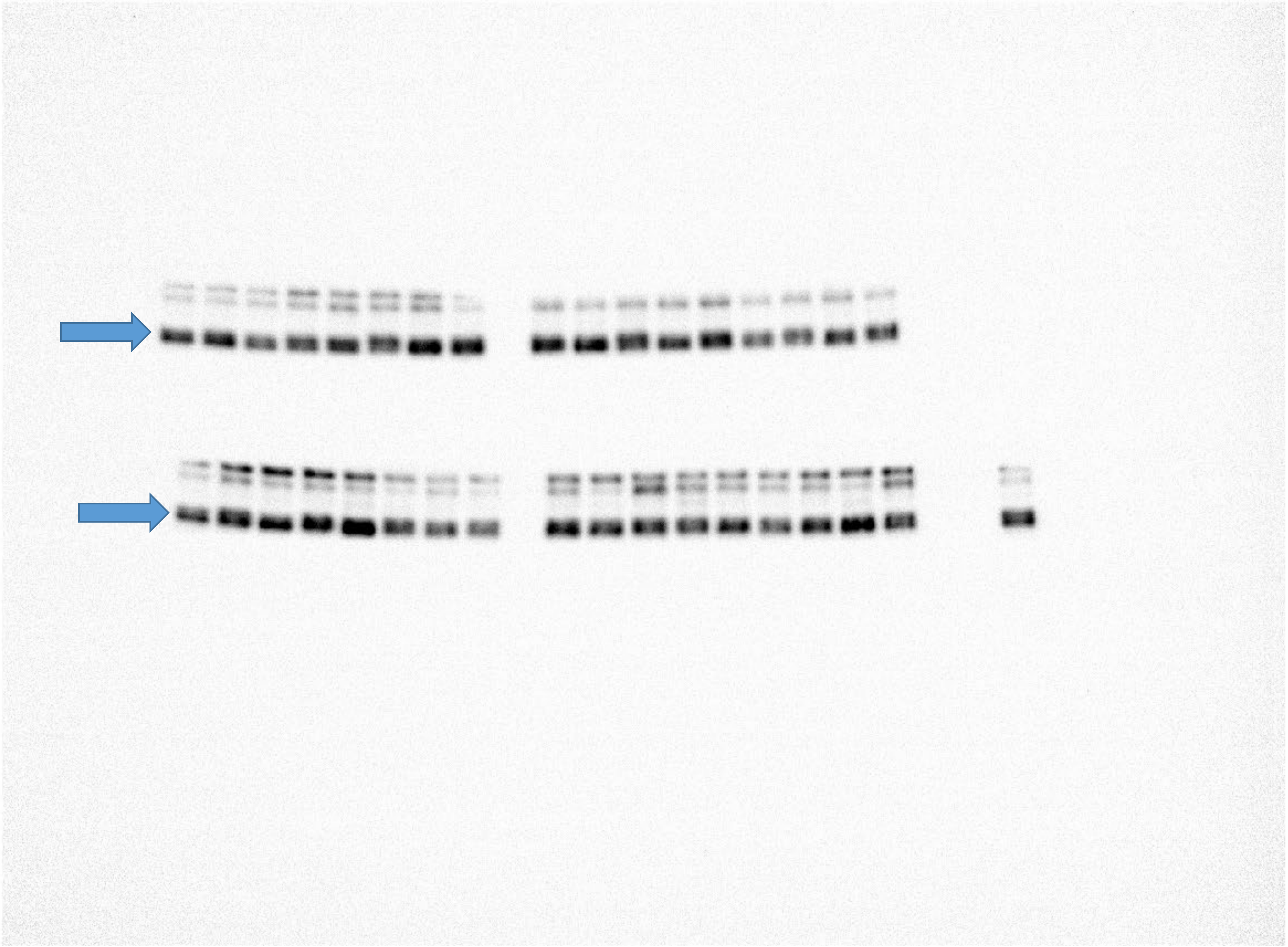

pmTOR_raw_image

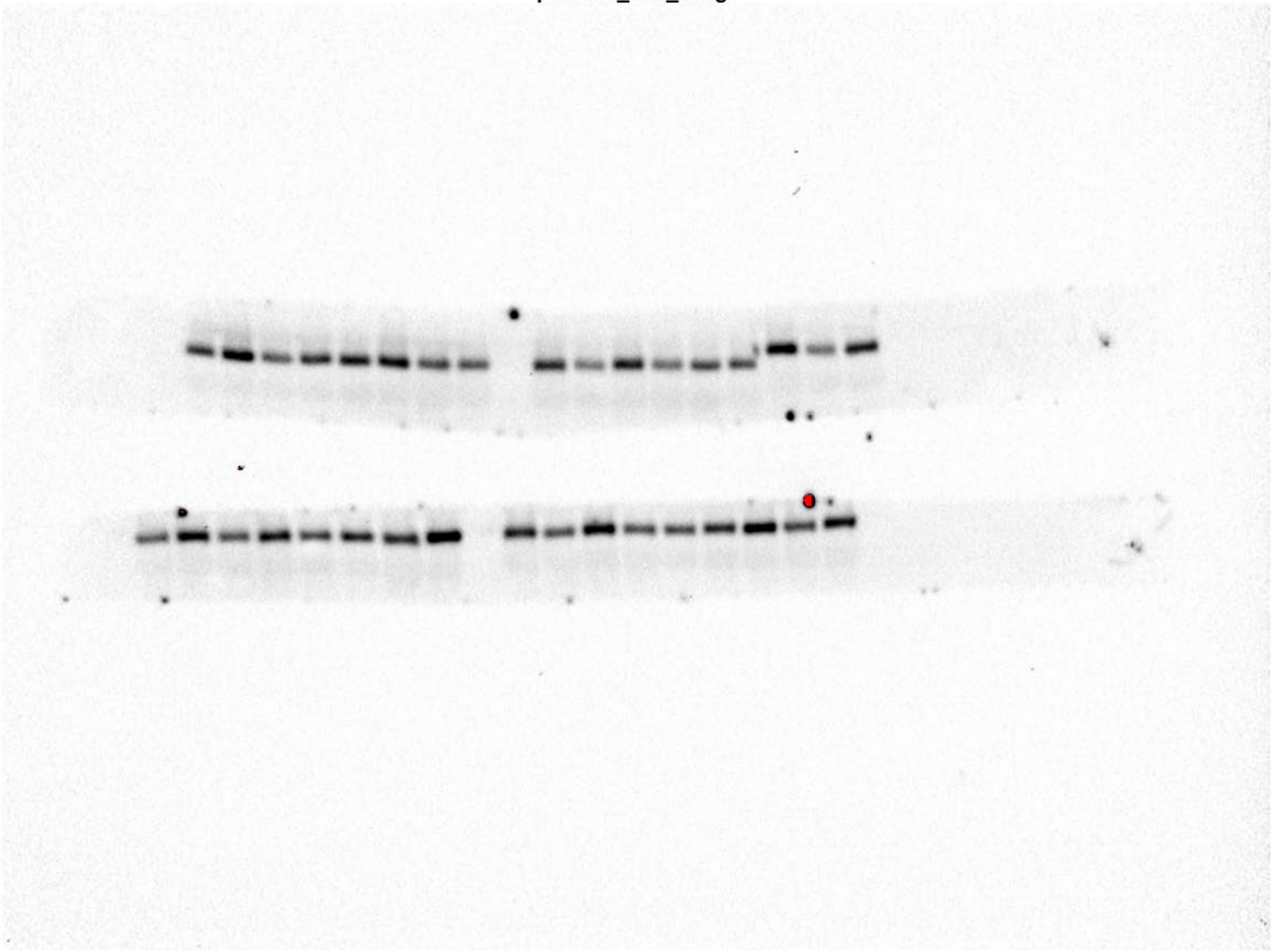

mTOR_raw_image

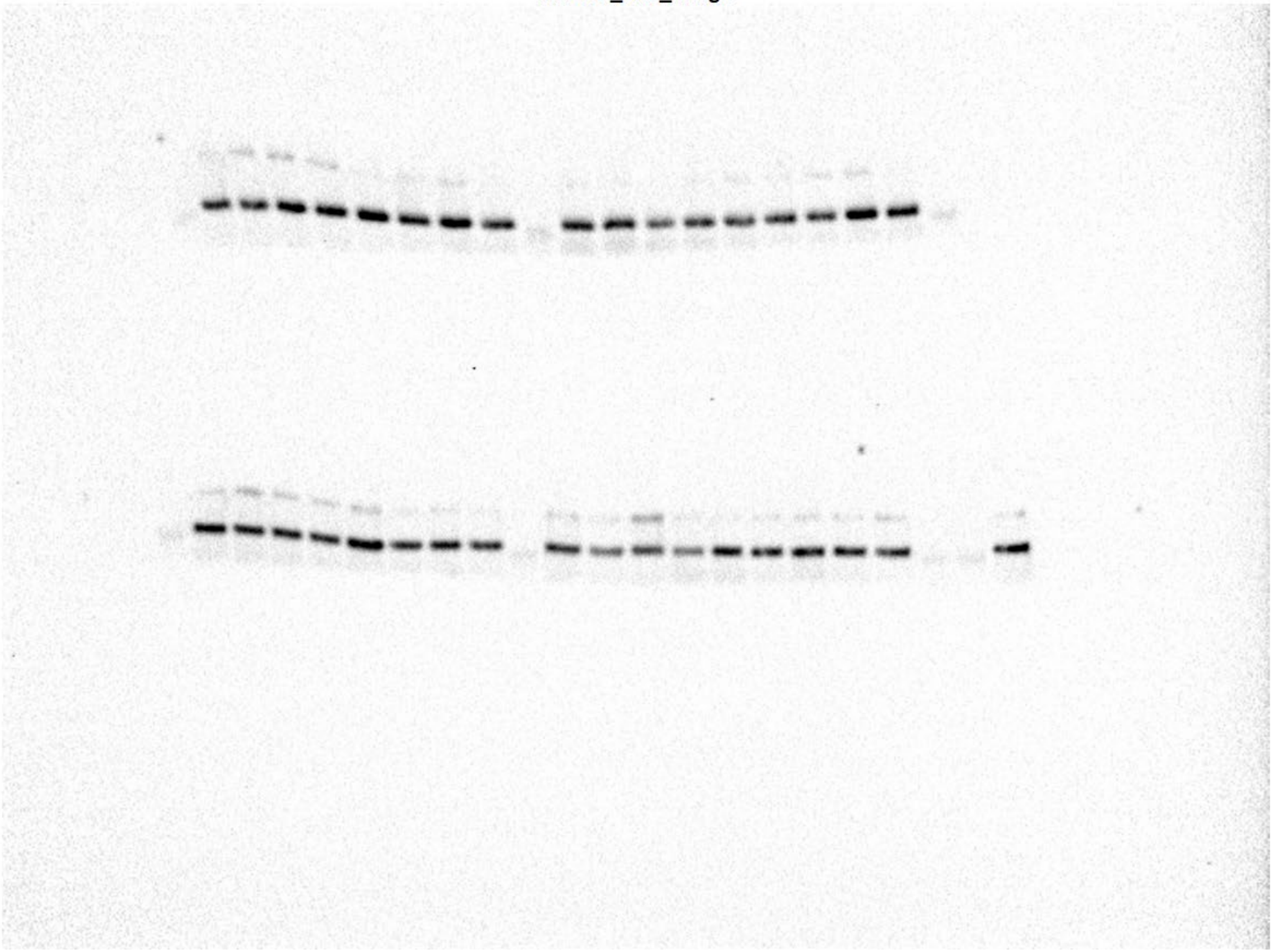

pS6_raw_image

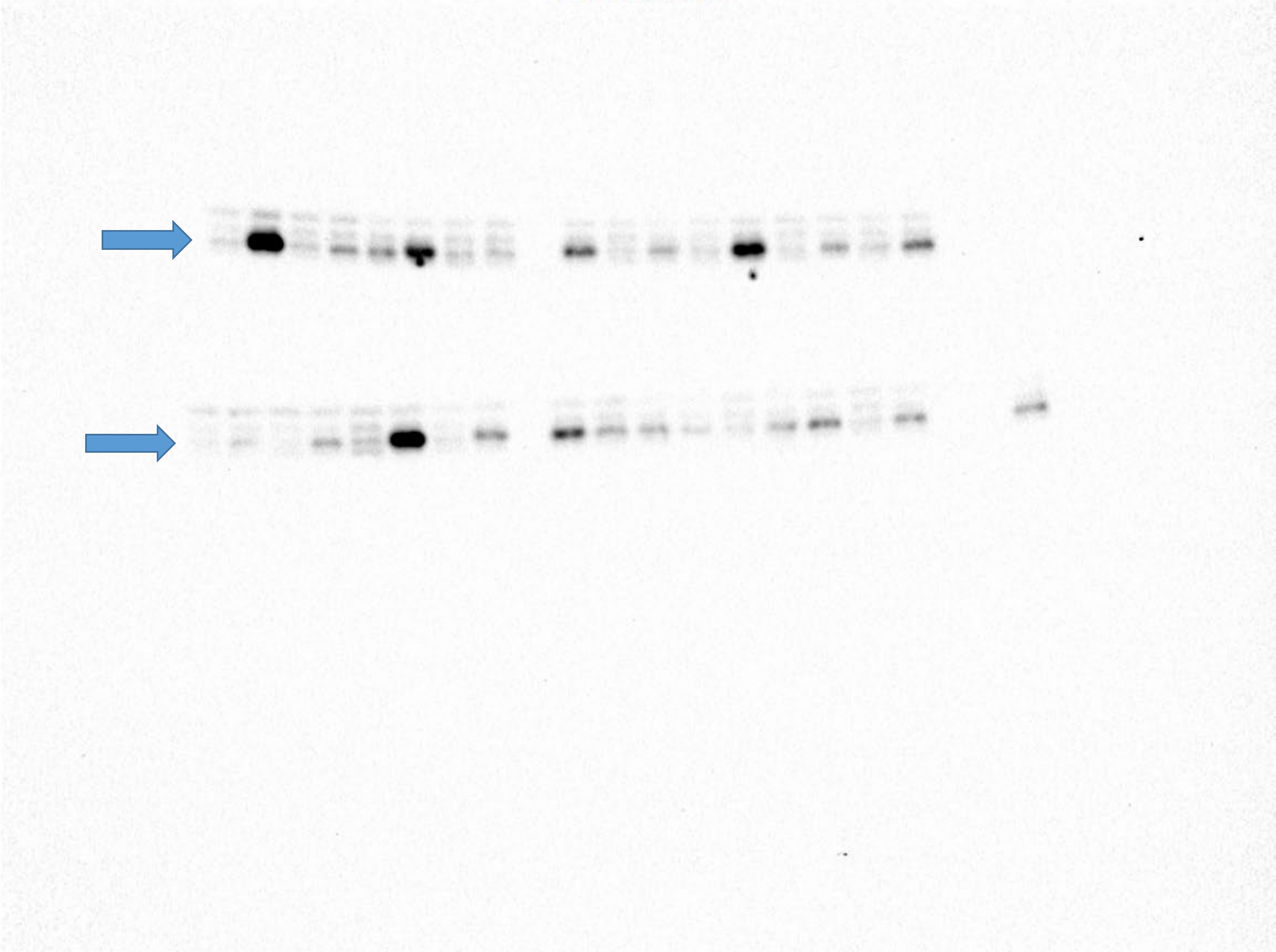

S6_raw_image

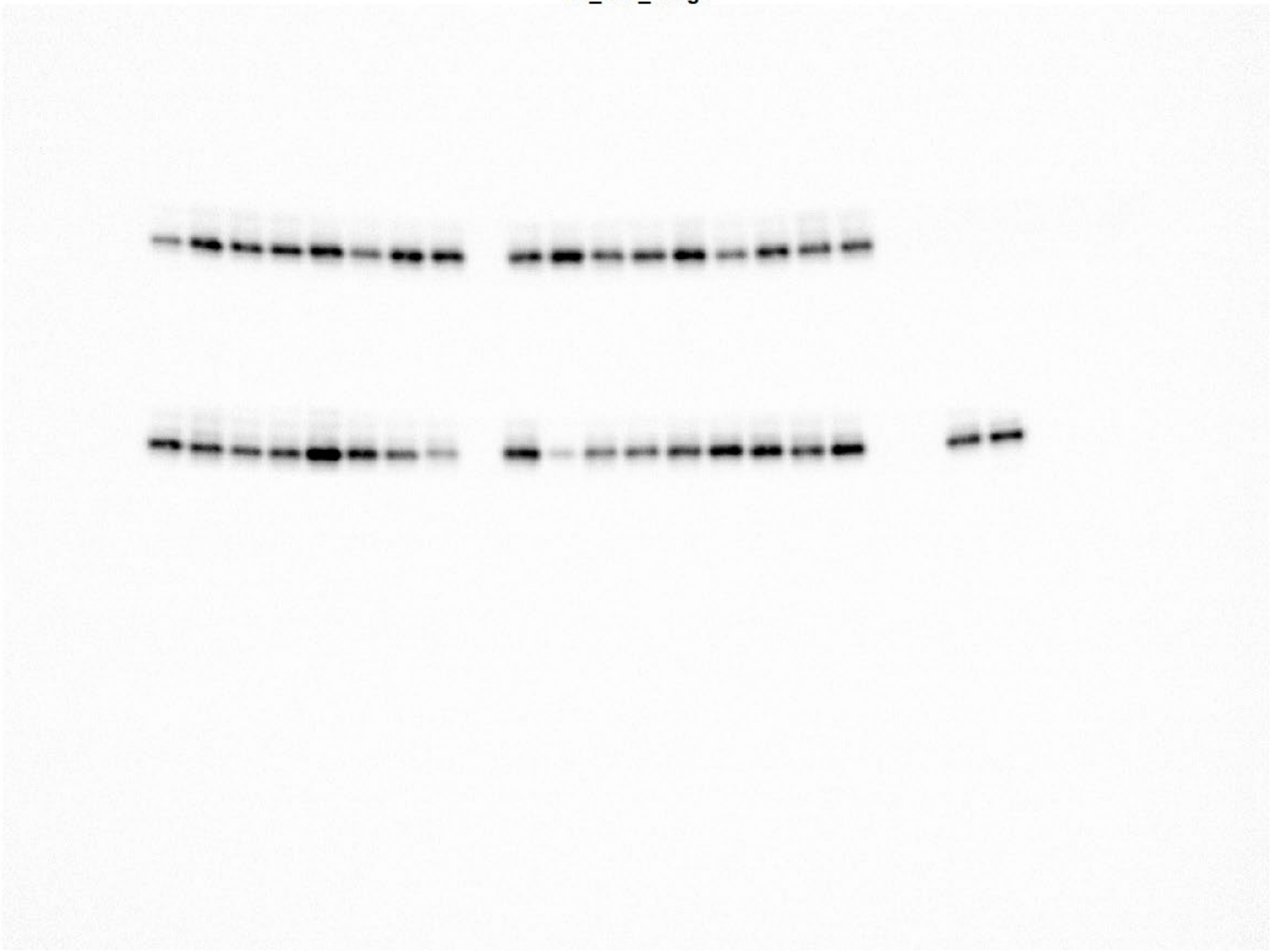

Non_p_4EBP1_raw_image

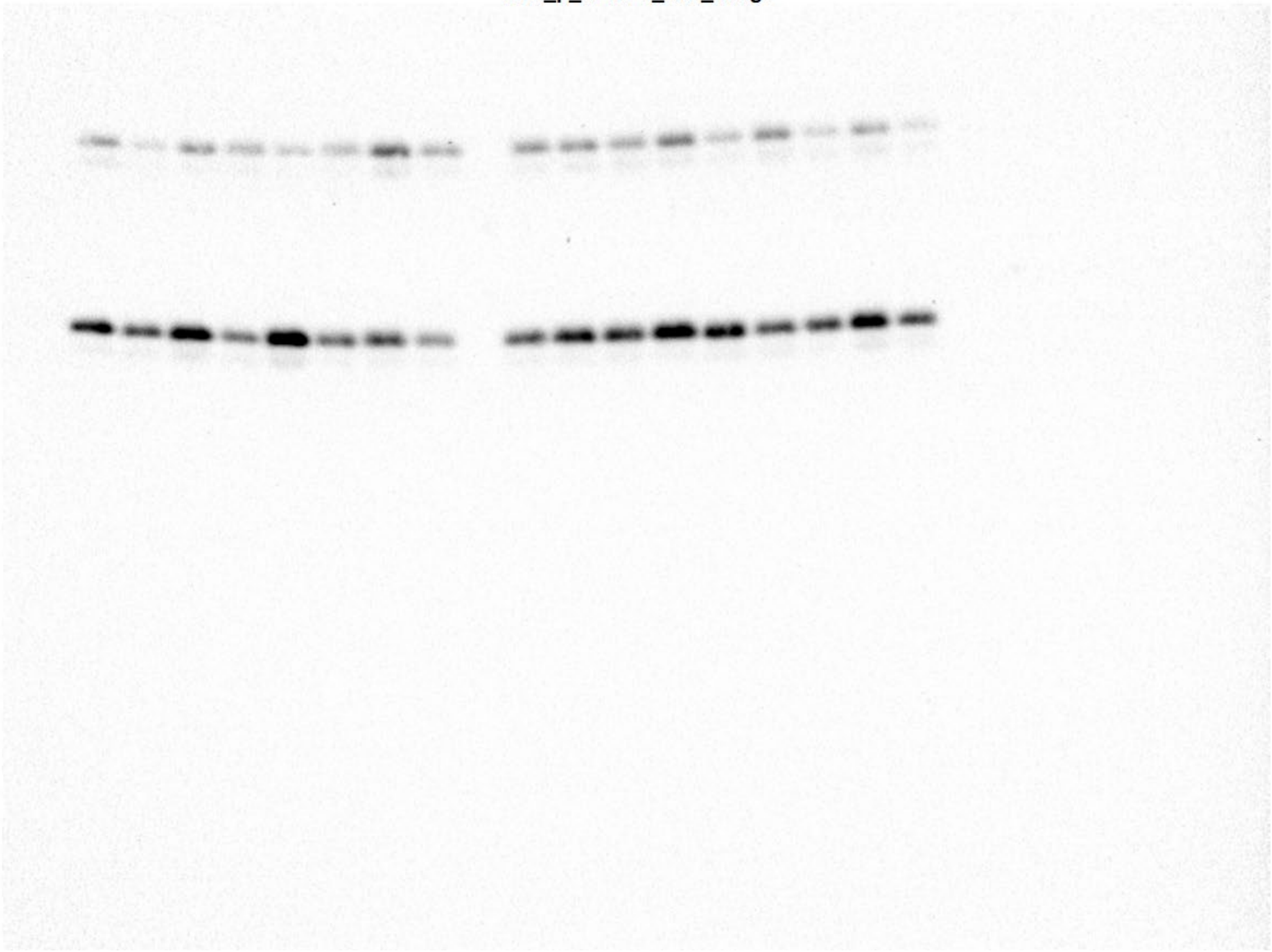

4EBP1_raw_image

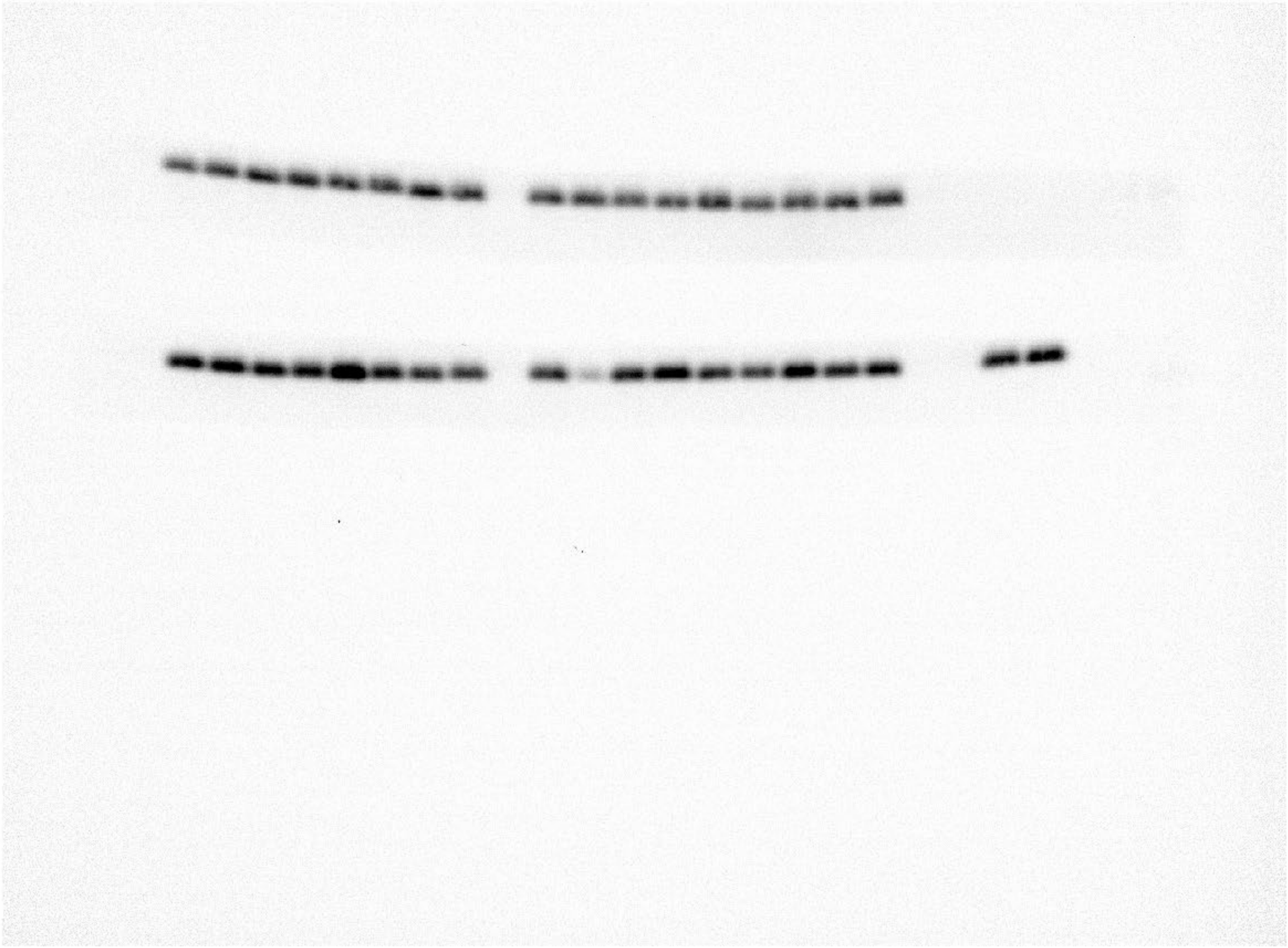

pULK555_raw_image

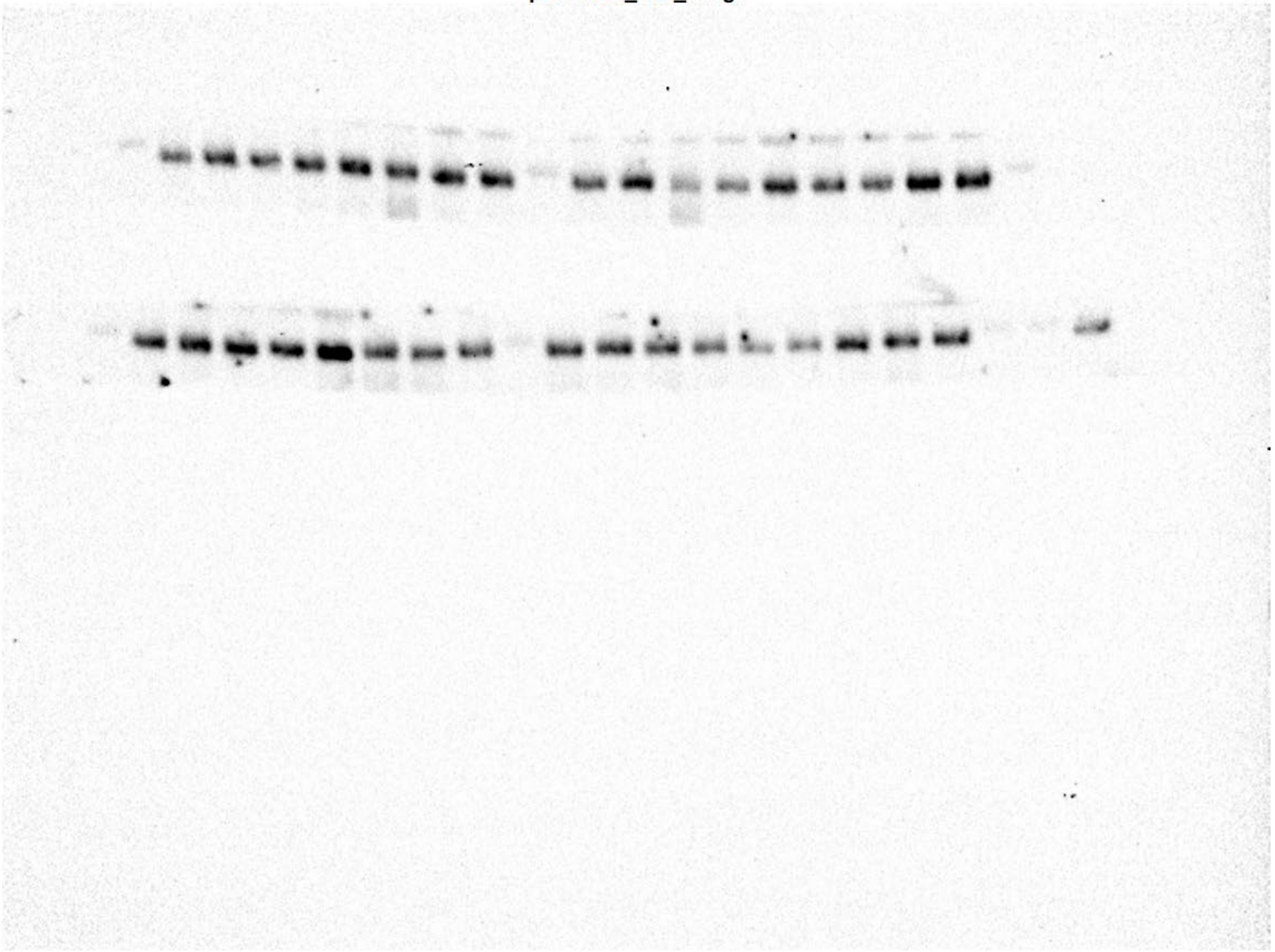

pULK757_raw_image

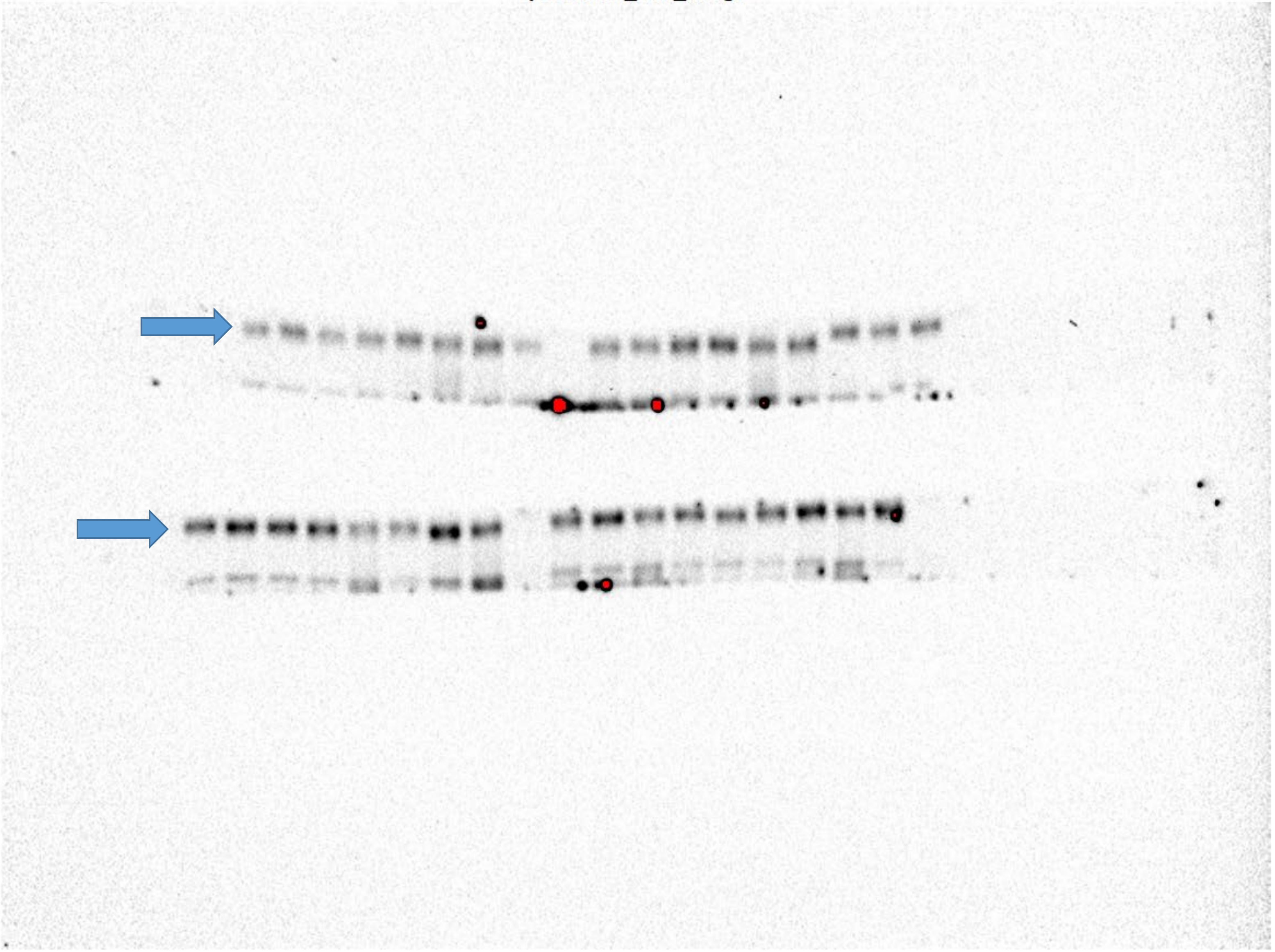

ULK1_raw_image

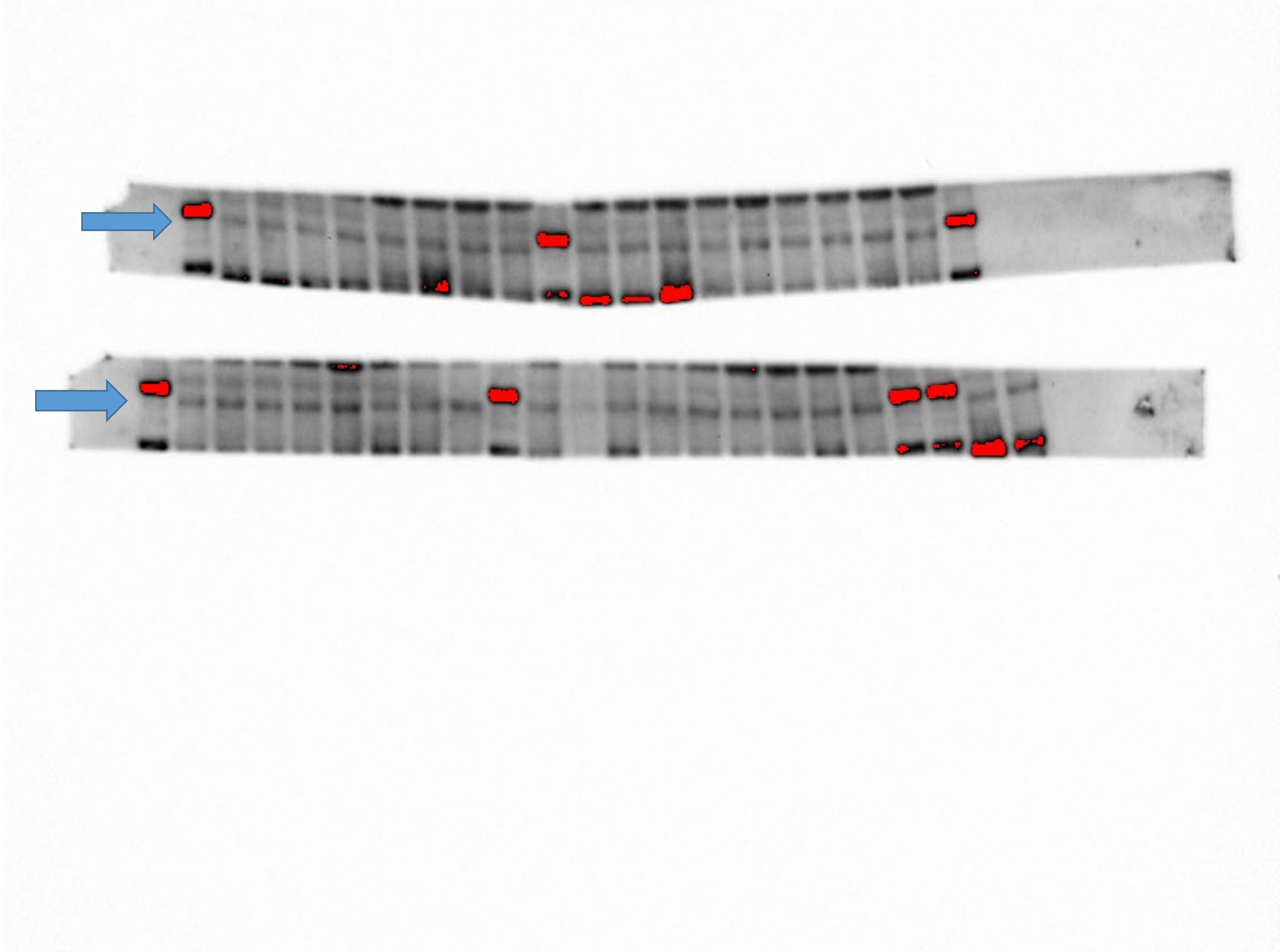

Fxb32_raw_image

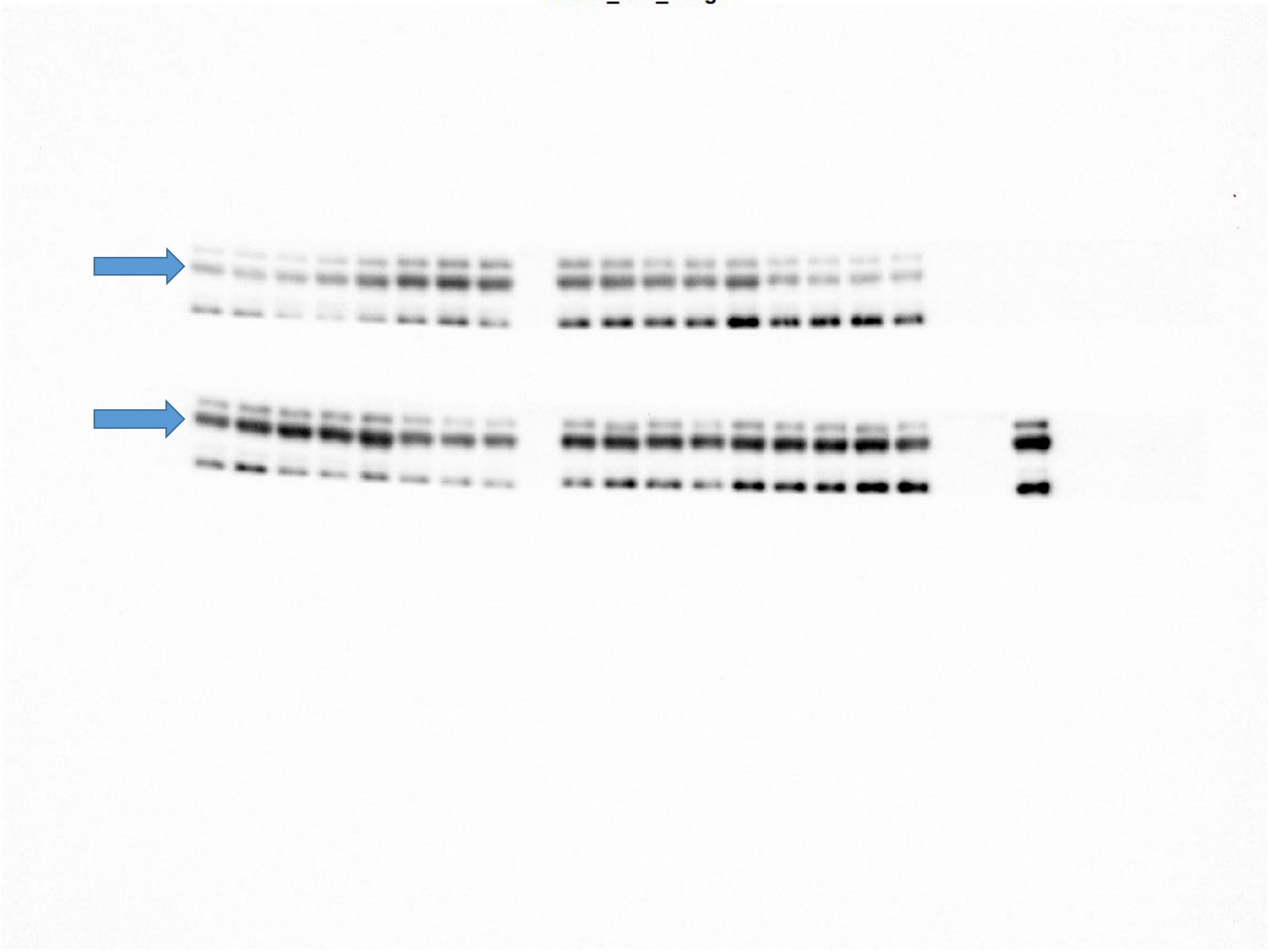

MURF1_raw_image

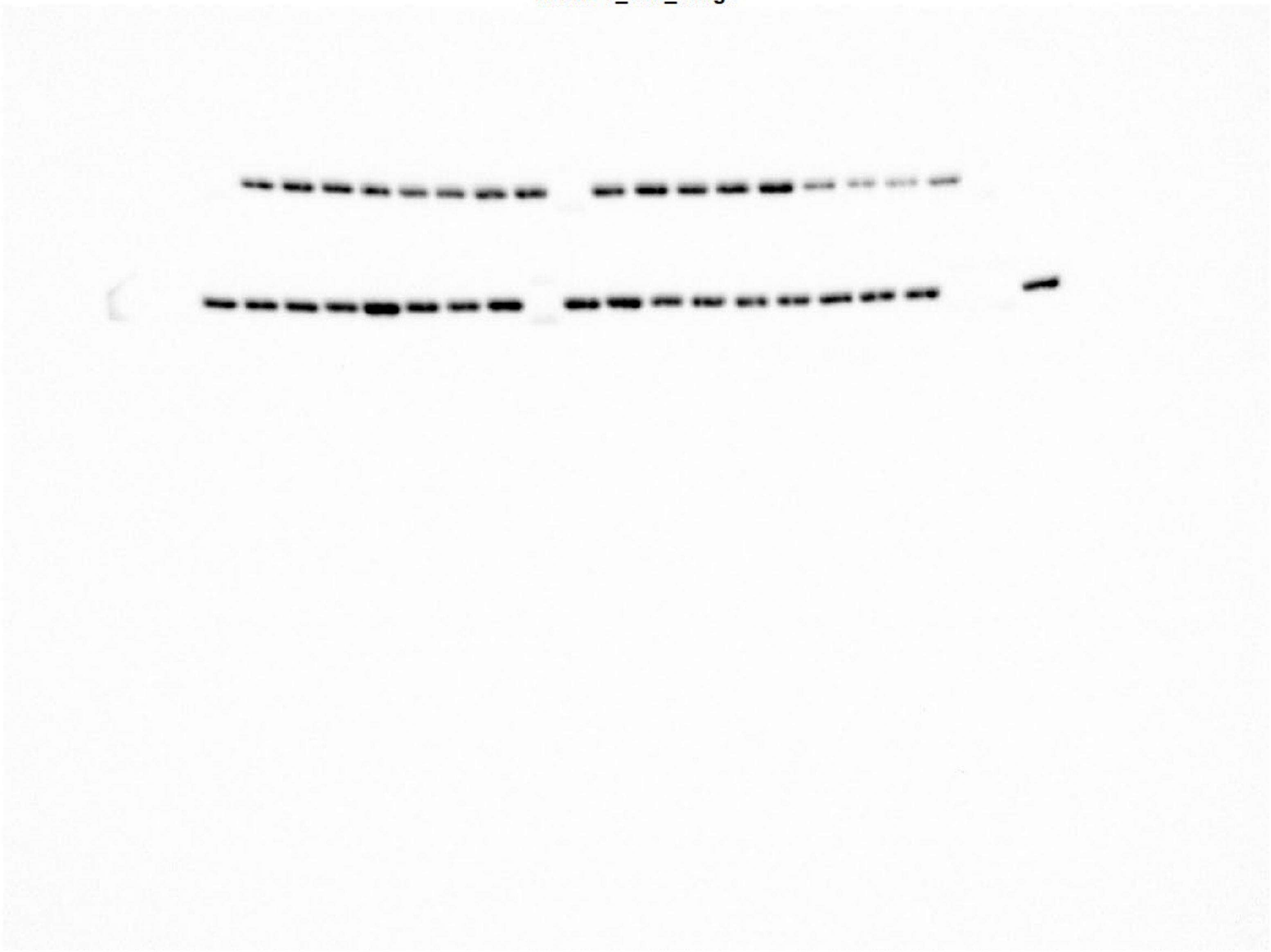

p62_raw_image

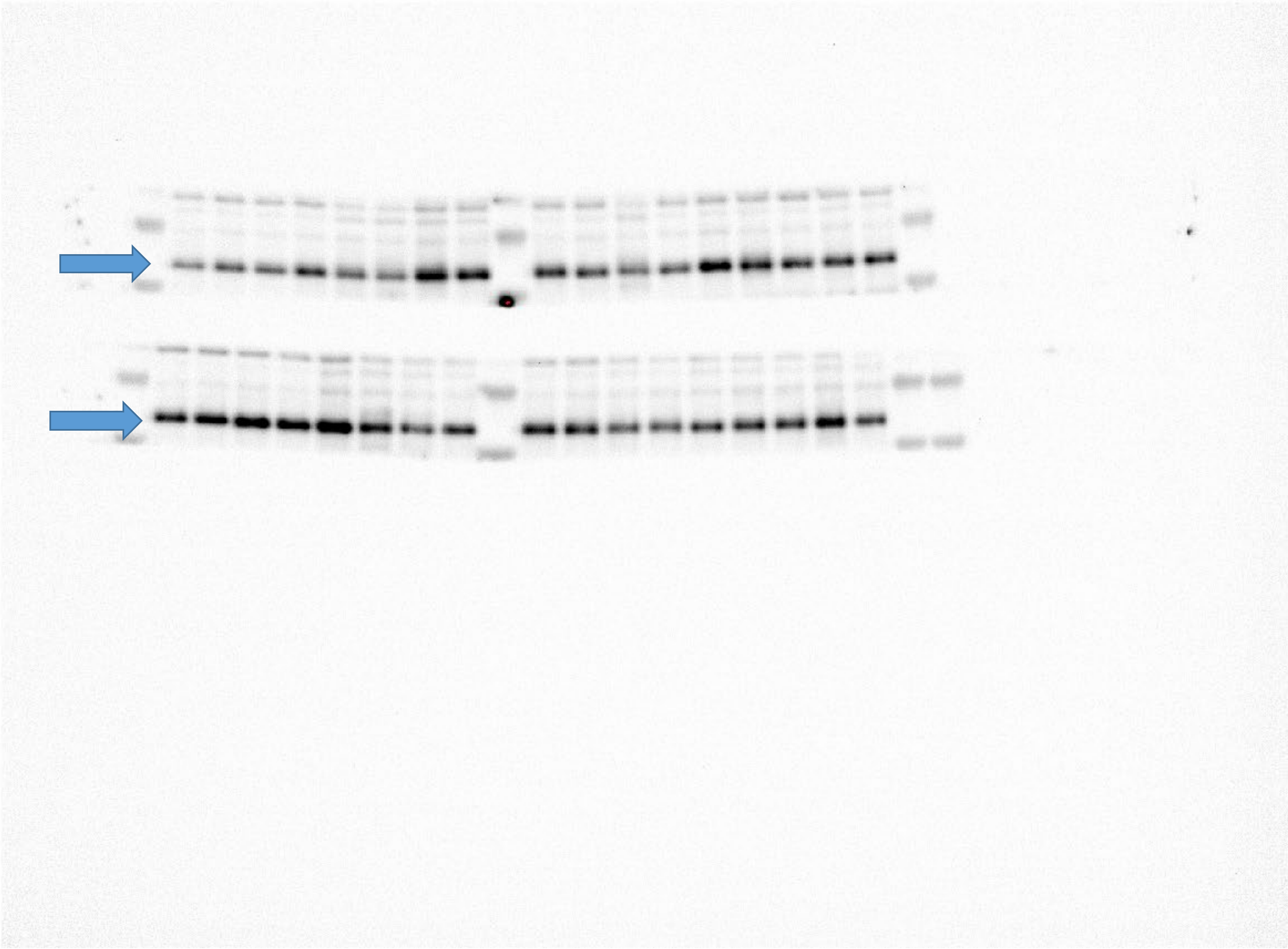

Supplement: S1 Raw images — (PDF) [file pone.0241274.s002.pdf]
